# Reassessing of the incremental predictive value of triglyceride-glucose index beyond traditional cardiovascular risk factors: a cohort study based on the China health and nutrition survey and meta-analysis

**DOI:** 10.3389/fcvm.2026.1804411

**Published:** 2026-07-22

**Authors:** Fang Liu, Zhipeng Zhao, Haidan Zhao

**Affiliations:** Department of Nephrology, Peking University Shougang Hospital, Peking, China

**Keywords:** cardiovascular disease, framingham risk score, insulin resistance, meta analysis, triglyceride-glucose index

## Abstract

**Background:**

This study aims to explore whether incorporating the triglyceride-glucose (TyG) index into the Framingham Risk Score (FRS) can enhance discriminative performance using raw data from the China Health and Nutrition Survey (CHNS) cohort, and to systematically quantify its incremental predictive value through a meta-analysis.

**Methods:**

Participants were adults aged 45 to 80 years without baseline cardiovascular disease (CVD). Incident CVD was defined as new diagnoses of myocardial infarction or stroke. Cox regression was used to examine the association between TyG and CVD, and the results were expressed as hazard ratios (HR) with 95% confidence intervals (CI). While the incremental predictive value of TyG added to FRS was assessed via C-index, integrated discrimination improvement (IDI) and net reclassification improvement (NRI). Restricted cubic splines in Poisson models explored non-linearity. For the meta-analysis, we included effect estimates for the incremental value of the TyG index.

**Results:**

The highest TyG quartile was significantly associated with higher risks of CVD (HR = 2.073; 95% CI: 1.233 to 3.488) and stroke (HR = 2.078; 95% CI: 1.428 to 5.521), with a non-linear dose-response relationship (*P*-nonlinear < 0.001). However, at the level of incremental predictive value, adding continuous TyG to FRS yielded a minimal C-index increase (from 0.731 to 0.732; *P* = 0.559). Adding TyG quartiles improved some reclassification metrics (IDI and NRI) despite no significant C-index gain. Subgroup analyses showed no significant interactions. For the meta-analysis, Pooling five cohort studies (CVD incidence: 3.3% to 14.4%) demonstrated a minimal overall C-index gain of 0.0004 (95% CI: −0.0092 to 0.0100). Sensitivity analysis revealed that this result was heavily influenced by the Niloofar Barzegar 2020; its exclusion increased the pooled C-index gain to 0.0051 (95% CI: 0.0007 to 0.0094). According to the Grading of Recommendations Assessment, Development, and Evaluation framework, the overall certainty of evidence for the incremental C-index change was rated as very low.

**Conclusions:**

While the TyG index shows a significant statistical association with CVD risk, its incremental predictive value beyond established FRS factors is minimal at the cohort level and negligible at the meta-analytic level. These findings suggest that the TyG index, despite being a significant risk marker, may not materially improve the predictive performance of existing cardiovascular risk scores.

## 1 Introduction

Cardiovascular disease (CVD), a major global public health concern, encompasses coronary heart disease (CHD), heart failure (HF), and stroke, etc. Data from the National Health and Nutrition Examination Survey between 2011and 2020 revealed an overall CVD prevalence of 9.6% among adults aged 30 to 79 years (1), posing substantial and unneglectable health threats and economic burdens (2). Early identification of CVD through population screening is crucial for optimizing the allocation of medical resources and improving patient prognosis.

Current CVD risk assessment mainly utilizes traditional tools, such as the well-established Framingham Risk Score (FRS) (3, 4) and pooled cohort equations (PCE) (5), which integrates key factors including age, blood pressure, and blood lipids, etc. However, advances in cardiovascular research have led to the identification of novel biomarkers. Their incorporation into risk assessment systems holds promise for enhancing prediction accuracy and clinical utility. Given that risk assessment directly informs primary prevention strategies (e.g., aspirin and statin use) (6–8), refining these tools is critical to improving CVD outcomes.

Insulin resistance (IR), a fundamental pathophysiological mechanism in CVD, is characterized by reduced sensitivity of target organs to the hormone (9). It accelerates atherosclerosis via multiple pathways, such as inducing inflammation, impairing endothelial function, promoting dyslipidemia, and increasing blood pressure (9). The triglyceride-glucose (TyG) index, calculated from fasting triglyceride and glucose levels, demonstrates a significant linear correlation with IR severity (10). Owing to its convenience and cost-effectiveness, the TyG index serves as a reliable surrogate marker for assessing IR (11). Multiple large-scale clinical studies have established a significant association between the TyG index and CVD incidence and mortality risk (12, 13). For instance, a prospective study of 403,335 individuals free of baseline CVD demonstrated that a high TyG index independently predicted CVD risk even after adjustment for traditional risk factors (14).

Although the predictive value of the TyG index for CVD has been preliminarily established, its incremental contribution to risk prediction performance when added to traditional cardiovascular factors—specifically regarding predictive accuracy, discrimination, and reclassification—remains to be systematically evaluated. Therefore, utilizing large-scale cohort data from the China Health and Nutrition Survey (CHNS), this study aims to compare the predictive performance of the FRS and an enhanced model integrating the TyG index with FRS. Furthermore, a meta-analysis will be conducted to comprehensively quantify the clinical utility of TyG index plus a standard CVD risk calculator, thereby providing new evidence for refining precise CVD risk assessment.

## 2 Methods

### 2.1 Study design and participants of China health and nutrition survey

This study utilized data from CHNS, which employs a multistage random cluster sampling strategy to establish a community-based longitudinal open cohort enrolling over 12,000 participants across 12 provinces in mainland China. Across its survey waves, the response rates ranged from 79% to 94% at the household level and 80% to 88% at the individual level. All participants provided written informed consent, with ethical approval granted by the Institutional Review Committees of the University of North Carolina at Chapel Hill, the National Institute for Nutrition and Health, and the Chinese Center for Disease Control and Prevention (approval code: No. 201524). The detailed CHNS protocol has been published previously (15, 16).

#### 2.1.1 Participants

Given that only laboratory data from the 2009 survey were publicly available, we selected this year as the baseline. Participants aged 45 to 80 years (*n* = 5,386) were initially eligible. We excluded 21 individuals with missing TyG index data and 206 with baseline CVD history, resulting in 5,159 participants at baseline. Subsequently, during the 2011 and 2015 follow-up surveys, we excluded an additional 494 participants due to missing follow-up data, yielding a final analytical cohort of 4,665 participants (Supplementary Figure S1).

#### 2.1.2 Outcome definition and follow-up

Reflecting its open-cohort design, CHNS participants could enter or exit at any survey round, and those lost to follow-up were eligible to re-enter later. We defined incident CVD events as new diagnoses of myocardial infarction (MI) or stroke (17, 18) occurring between 2009 and 2015. Diagnostic information was collected via questionnaire interviews, relying on self-reported or proxy-reported physician diagnoses. Baseline CVD status was ascertained from the 2009 survey data, while incident CVD events were identified during the 2011 and 2015 follow-up surveys (19). Follow-up commenced on the date of the 2009 survey. For participants experiencing a CVD event, follow-up ended at the date of first diagnosis; for non-cases, it ended at the date of their last survey participation.

#### 2.1.3 Collection of covariates

Covariates were obtained from structured questionnaires administered during the 2009 baseline survey, encompassing: Demographic indicators included age, gender, and educational level (primary school and lower, junior and senior middle school, college and higher). Lifestyle factors involved smoking status (current smoker, quit smoker and never smoker) and drinking status (current drinker and non-drinker). Disease history was collected via self-report, including hypertension and antihypertensive medication use, diabetes and antidiabetic medication use, myocardial infarction, and stroke. Body mass index (BMI) was calculated using the measured weight and height data at baseline. Systolic blood pressure (SBP) and diastolic blood pressure (DBP) were determined as the mean values of three measurements taken in a resting state. Laboratory data were obtained from fasting blood tests, including fasting blood glucose (FBG), glycated hemoglobin (HbA1c), triglycerides (TG), total cholesterol (TC), high-density lipoprotein cholesterol (HDL-C), low-density lipoprotein cholesterol (LDL-C), C-reactive protein, and creatinine.

#### 2.1.4 Statistical analysis

Participants were categorized into quartiles based on the TyG index. Baseline characteristics are summarized as median (interquartile range) for continuous variables and count (percentage) for categorical variables. Intergroup differences were assessed using the Mann–Whitney *U* test for continuous variables and the chi-square test for categorical variables. Missing data (1.38% overall) were handled using multiple imputation by chained equations via the mice package, under the missing-at-random assumption. The imputation model included all baseline covariates listed in Table 1, the outcome indicator (incident CVD), and the follow-up time to preserve the event–covariate relationship for survival data. A total of 20 imputed datasets were generated with 10 iterations each; convergence was confirmed by visual inspection of trace plots. Continuous variables were imputed using predictive mean matching, and categorical variables using logistic regression (20). First, Cox proportional hazards regression models were employed to examine the association of the TyG index with incident CVD. Multivariable-adjusted hazard ratios comparing the highest vs. lowest TyG quartiles were calculated. Adjustment covariates comprised the FRS factors: age, sex, SBP, antihypertensive treatment, TC, HDL-C, current smoking status, and diabetes.

**Table 1:** Characteristics of TyG index tertiles in the participants (*n* = 4,665).

Variables	Overall (*n* = 4,665)	CVD (*n* = 210)	Without CVD (*n* = 4,455)	*P*
Male sex, *n* (%)	2,168, (46.47%)	112, (51.33%)	2,056, (46.15%)	0.041
Age (years)	57.0 (51.0 to 64.0)	63.0 (56.5 to 70.0)	57.0 (51.0 to 64.0)	<0.001
BMI, (kg/m^2^)	23.45 (21.30 to 25.76)	24.81 (22.12 to 26.68)	23.39 (21.26 to 25.73)	<0.001
Tobacco use				
Never, *n* (%)	3,151, (67.56%)	139, (66.19%)	3,012, (67.61%)	0.668
Current, *n* (%)	1,342, (28.77%)	57, (27.14%)	1,285, (28.84%)	0.595
Quit, *n* (%)	172, (3.69%)	14, (6.67%)	158, (3.55%)	0.019
Alcohol consumption, *n* (%)	1,512, (32.41%)	63, (30.00%)	1,449, (32.53%)	0.445
SBP (mmHg)	126.67 (118.00 to 140.00)	140.00 (124.00 to 156.33)	126.00 (117.33 to 139.33)	<0.001
DBP (mmHg)	80.67 (76.00 to 90.00)	85.00 (80.00 to 92.00)	80.67 (76.00 to 89.33)	<0.001
Educational level				
Primary school and lower, *n* (%)	2,496, (53.50%)	139, (66.19%)	2,357 (52.91%)	<0.001
Junior and senior middle school, *n* (%)	2,024, (43.39%)	66, (31.43%)	1,958, (43.95%)	<0.001
College and higher, *n* (%)	145, (3.11%)	5, (2.38%)	140, (3.14%)	0.534
Comorbidities				
Hypertension, *n* (%)	1,619, (34.71%)	132, (62.86%)	1,487, (33.38%)	<0.001
Treatment for hypertension, *n* (%)	634, (13.59%)	73, (34.76%)	561, (12.59%)	<0.001
Diabetes, *n* (%)	507, (10.87%)	31, (14.76%)	476, (10.68%)	0.064
Treatment for diabetes, *n* (%)	154, (3.30%)	16, (7.62%)	138, (3.10%)	<0.001
Laboratory parameters				
FBG, (mmol/L)	5.23 (4.80 to 5.74)	5.36 (4.88 to 5.81)	5.22 (4.79 to 5.74)	0.028
Hba1c, (%)	5.6 (5.3 to 5.9)	5.7 (5.4 to 6.1)	5.6 (5.3 to 5.9)	0.001
TG, (mmol/L)	1.34 (0.91 to 2.06)	1.50 (1.07 to 2.22)	1.34 (0.90 to 2.06)	0.002
TC, (mmol/L)	4.96 (4.35 to 5.65)	4.89 (4.36 to 5.53)	4.52 (4.03 to 5.07)	0.055
HDL to C, (mmol/L)	54.14 (45.24 to 64.19)	51.82 (43.12 to 62.45)	54.52 (45.63 to 64.29)	0.015
LDL to C, (mmol/L)	118.33 (95.90 to 142.69)	124.13 (103.05 to 147.33)	117.94 (95.51 to 142.69)	0.009
TyG index	8.64 (8.22 to 9.13)	8.79 (8.41 to 9.22)	8.63 (8.21 to 9.12)	0.001

Values are *n* (%), mean ± standard deviation or median (interquartile range). BMI, body mass index; SBP, systolic blood pressure; DBP, diastolic blood pressure; FBG, fasting blood glucose; TG, triglyceride; TC, total cholesterol; HDL-C, high-density lipoprotein cholesterol; LDL-C, low-density lipoprotein cholesterol; TyG, triglyceride-glucose. Missing data: 3 for tobacco use; 1for alcohol consumption; 8 for educational level; 76 for BMI; 28 for HbA1c; 64 for SBP.

Second, we evaluated the incremental predictive value by comparing: base model containing only FRS variables; Model 1 defined as base model with continuous TyG index; Model 2 including base model and TyG index quartiles. Model discrimination was assessed using the concordance statistic (C-index), which quantifies the ability to differentiate between individuals who do and do not experience the event. The statistical significance of differences in C-index was evaluated with the DeLong test. Additionally, we estimated the correlation coefficient (*r*) for each model using 1,000 bootstrap samples (21). Next, the Net Reclassification Improvement (NRI) (22) was calculated to quantify the improvement in correct reclassification of events and non-events achieved by adding the continuous TyG index to the FRS. Participants were stratified into low-risk (<10%), intermediate-risk (10% to <20%), and high-risk (≥20%) (3, 23) categories based on predicted risk of 3 models. The Integrated Discrimination Improvement (IDI) (22) was used to evaluate whether the new models provided superior discrimination compared to the base model.

To investigate potential non-linearity, restricted cubic splines (RCS) within Poisson regression models (24) were applied to examine the relationship between the TyG index and CVD probability. The optimal number of knots (tested between 3 and 7) was chosen based on the Akaike Information Criterion.

Finally, participants were reclassified into low-, intermediate-, and high-risk groups using the base model and model 1. Subgroup-specific restricted cubic spline plots (based on Poisson regression) were generated to visualize associations within each risk stratum. Statistical evidence for interaction was assessed using the likelihood ratio test, yielding a *P*-interaction value. Subgroup analyses were conducted for both the continuous TyG index and TyG index quartiles, stratified by: age, sex, body mass index, smoking history, alcohol consumption history, hypertension (history and treatment), diabetes (history and treatment), and FRS risk category.

All statistical analyses were conducted using R software (version 4.3.3; R Foundation for Statistical Computing, Vienna, Austria). A two-sided *P*-value <0.05 defined statistical significance.

### 2.2 Systematic review and meta-analysis

This systematic review adheres to the Meta-analysis of Observational Studies in Epidemiology reporting guidelines and is registered on PROSPERO (CRD42024525061).

#### 2.2.1 Data sources and search strategy

We systematically searched PubMed, Web of Science, and Embase up to August 22, 2025. Following deduplication, two independent reviewers (Fang Liu and Zhipeng Zhao) screened titles and abstracts, then assessed full texts of potentially eligible articles against the inclusion criteria. A third reviewer (Haidan Zhao) resolved disagreements. The screening process is detailed in Supplementary Figure S2.

#### 2.2.2 Inclusion criteria

We included cohort studies that assessed the incremental value of adding the TyG index to conventional CVD risk prediction models, provided they met these criteria: Study participants were community-dwelling individuals or outpatients without baseline CVD; National guideline-recommended CVD risk calculators (e.g., FRS, PCE) were used; the outcome of the risk prediction model was cardiovascular events. The PICO criteria were specifically defined as follows: Participants were adult free of CVD (e.g., the general population); Intervention consisted of TyG index combined with a CVD risk prediction model; Comparison consisted of CVD risk prediction model alone; outcomes consisted of Incident CHD, stroke, or their composite; and study design consisted of Cohort studies reporting incident cardiovascular events. We excluded studies conducted exclusively in specific patient subgroups and those not published in English or Chinese.

#### 2.2.3 Data extraction

Two reviewers (Fang Liu and Zhipeng Zhao) independently extracted study characteristics, outcomes, and risk of bias data using a predefined form. Information on race and ethnicity was primarily self-reported.

#### 2.2.4 Risk of bias assessment

Two reviewers (Fang Liu and Zhipeng Zhao) independently assessed the risk of bias for each included study using the modified Quality In Prognosis Studies tool (25). They rated each domain (potential source of bias) as low, high, or unclear risk. Disagreements were resolved through consensus discussion. The assessment covered six domains of bias. First, study participation bias was evaluated based on the representativeness of the study sample. Second, study attrition bias was assessed according to the participants with available follow-up data. Third, bias in prognostic factor measurement focused on the measurement of TyG index. Fourth, bias in outcome measurement, namely the similarity of outcome assessment. Fifth, study confounding bias, particularly emphasized the potential confounding factors that were accounted for. Sixth, bias in statistical analysis and reporting referred to the appropriateness of statistical methods and reporting.

#### 2.2.5 Meta-analysis

We conducted a meta-analysis of the difference in C-index between the enhanced model (incorporating the TyG index + traditional model) and the traditional model alone. For each study, the 95% CI for the C-index difference was either extracted directly or derived by calculating the standard error (SE) of the difference between two correlated C-indices. Specifically, we applied the method proposed by Hanley and McNeil (1983), which computes the SE of the difference between two correlated C-index derived from the same sample (26). For studies that did not report this correlation coefficient, we approximated it using the correlation coefficient empirically derived from our CHNS cohort, which provided the necessary parameter to complete the meta-analysis. The statistical significance of differences in C-statistics was evaluated using the DeLong test. We assessed statistical heterogeneity using Cochran's *Q* test and quantified it with the *I*^2^ statistic. Summary estimates were pooled using a random-effects model. A forest plot illustrates the summary change in C-index along with study-specific estimates. Owing to the small number of included studies, we did not perform meta-regression, nor did we assess publication bias or small-study effects.

#### 2.2.6 Sensitivity and subgroup analyses

We performed sensitivity analysis using the leave-one-out method. Additionally, we conducted three subgroup analyses to estimate the improvement in the C-index: First, studies based on Chinese cohorts; Second, studies which the traditional model utilized FRS; Third, studies defining the outcome as CHD, stroke, and peripheral vascular disease. Planned subgroup analyses examining CHD events specifically, stroke events specifically, and stratification by baseline CVD risk levels (according to the traditional model) could not be performed due to insufficient data.

#### 2.2.7 GRADE certainty assessment

The certainty of evidence for the incremental predictive value of the TyG index was assessed using the GRADE (Grading of Recommendations Assessment, Development and Evaluation) framework. Evidence from observational cohort studies started at low certainty and was downgraded or upgraded based on five domains: risk of bias, inconsistency, indirectness, imprecision, and publication bias, as well as three upgrading factors (large effect size, dose-response gradient, and plausible confounding would reduce the effect). Two separate assessments were performed: one for the main meta-analysis including all five studies, and another for the sensitivity analysis excluding the outlier study (27).

## 3 Results

### 3.1 Findings from the China health and nutrition survey

The cohort comprised 4,665 participants, including 2,186 (46.47%) males. Median age was 57.0 years (51.0 to 64.0). As detailed in Table 1, significant differences (all *P* < 0.05) were observed between participants who developed CVD and those who did not across multiple covariates: sex, age, BMI, smoking status, educational level, SBP, DBP, history of hypertension and antihypertensive medication use, antidiabetic medication use, FBG, TG, HDL-C, and LDL-C.

Over a median follow-up of 6 years, 210 participants (4.5%) developed CVD, 92 (2.0%) had MI and 126 (2.7%) had stroke. Hazard ratios for traditional risk factors associated with incident CVD in the base model are presented in Supplementary Table S1. Participants in the highest TyG index quartile had significantly higher risks of CVD (HR, 2.073; 95% CI, 1.233 to 3.488; *P* = 0.006) and stroke (HR, 2.078; 95% CI, 1.428 to 5.521; *P* = 0.002) compared to those in the lowest quartile CVD (Supplementary Table S2).

Table 2 summarizes the incremental predictive value of adding the TyG index (continuous or as quartiles) to the FRS for CVD events. Adding the continuous TyG index yielded a minimal increase in the C-index (from 0.731 [95% CI 0.698 to 0.764] to 0.732 [95% CI 0.699 to 0.765]; *P*-DeLong = 0.5585). Neither the IDI (0.0002, 95% CI 0.0001 to 0.0003; *P* > 0.05) nor the Continuous NRI (−0.0090, 95% CI −0.1465 to 0.1285; *P* > 0.05) showed significant improvement. Adding TyG index quartiles increased the C-index to 0.739 (95% CI 0.706 to 0.772; *P* for DeLong = 0.2136). Although the C-index improvement was not statistically significant by the DeLong test, significant enhancements were observed in both the IDI (0.0034, 95% CI 0.0005 to 0.0064; *P* < 0.05) and the Continuous NRI (0.2133, 95% CI 0.0981 to 0.3285; *P* < 0.05), indicating improved discrimination and reclassification. Results from Bootstrap analysis (1,000 replicates) revealed that the *r* between base model and model1 was 0.9976 (SE: 0.0012), with a change in C-index (ΔC) of 0.0010 (95% CI: −0.0014 to 0.0034). The *r* between the base model and model 2 was 0.9886 (SE: 0.0026), with a ΔC of 0.0080 (95% CI: 0.0029 to 0.0131).

**Table 2:** Comparison of the predictive value of TyG index and TyG quartiles for incident CVD.

CVD	C-index (95% CI)	*P*	IDI (95% CI)	*P*	Continuous NRI	*P*	ΔC (95% CI)	SE	*r*
Base model	0.7310 (0.6980 to 0.7640)	Ref	Ref	Ref	Ref	Ref	Ref	Ref	Ref
Model 1	0.7320 (0.6990 to 0.7650)	0.5585	0.0002 (0.0001 to 0.0003)	0.5445	−0.0090 (−0.1465 to 0.1285)	0.8978	0.0010 (−0.0014 to 0.0034)	0.0012	0.9976
Model 2	0.7390 (0.7060 to 0.7720)	0.2136	0.0034 (0.0005 to 0.0064)	0.0231	0.2133 (0.0981 to 0.3285)	0.0003	0.0080 (0.0029 to 0.0131)	0.0026	0.9886

TyG, triglyceride-glucose; CVD, cardiovascular disease; FRS, Framingham risk score; IDI, integrated discrimination improvement, NRI, net reclassification improvement; SE, standard error; *r*, correlation coefficient. Base model: adjusted for age, sex, systolic blood pressure, antihypertensive treatment status, total cholesterol, high-density lipoprotein cholesterol, current smoking status, and diabetes. Model 1: base model with continuous TyG index. Model 2: base model with TyG quartiles.

Stratified NRI analyses (Supplementary Tables S3, S4) by baseline risk category (low: ≤10%, intermediate: 10% to 20%, high: ≥20%) indicated that adding the continuous TyG index provided limited reclassification improvement. Changes in event NRI (−0.010; 95% CI −0.029 to 0.009; *P* > 0.05) and non-event NRI (0.001; 95% CI 0.001 to 0.004; *P* > 0.05) were non-significant. Adding TyG index quartiles significantly improved event reclassification (event NRI: 0.0476; 95% CI −0.0048 to 0.0913; *P* < 0.05) but not non-event reclassification (non-event NRI: −0.0007; 95% CI −0.0058 to 0.0045; *P* > 0.05).

**Figure 1:**
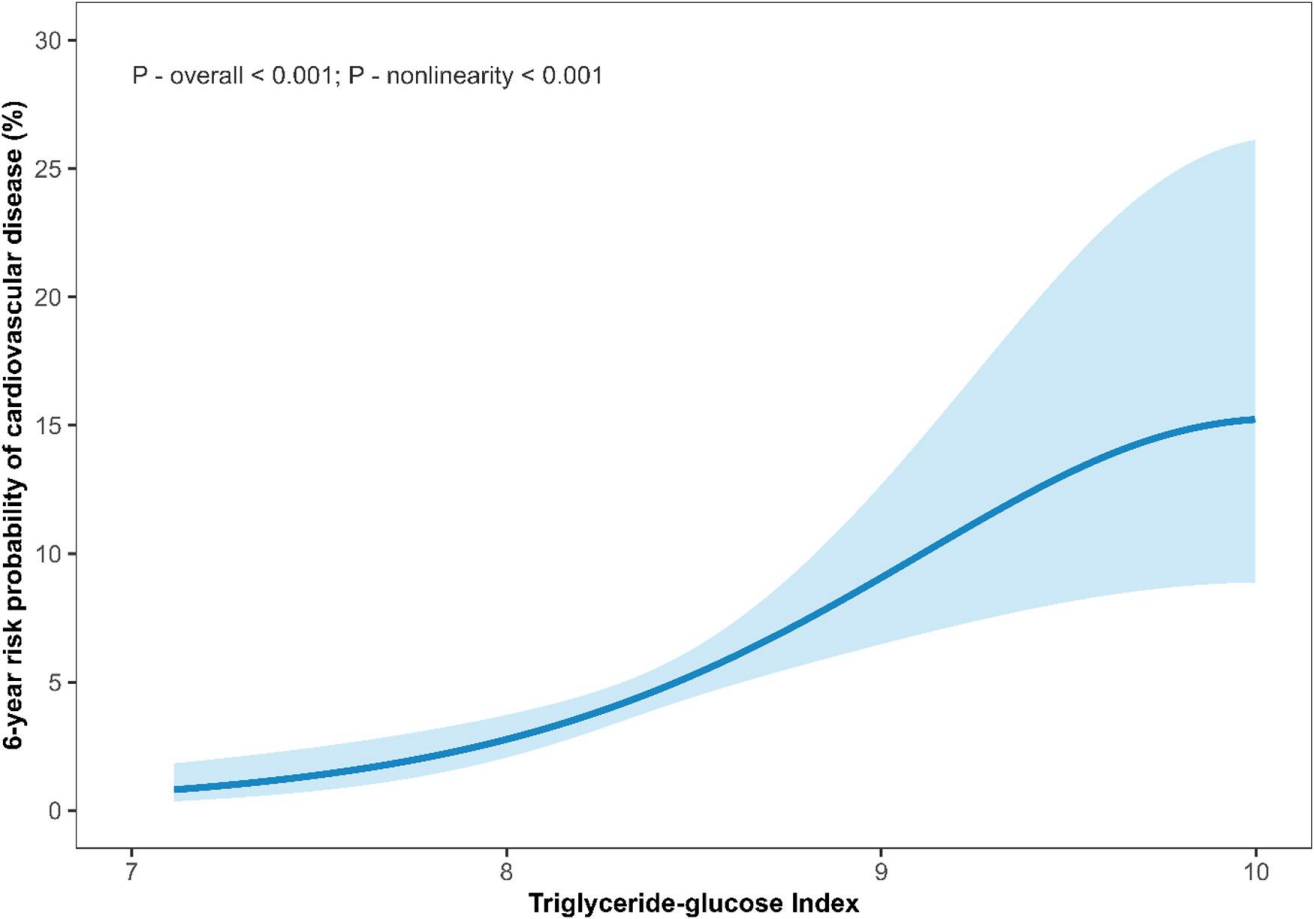
Flexible relationships between TyG index and risk probability of incident cardiovascular disease.

Figure 1 demonstrates a significant nonlinear dose-response relationship between the TyG index and the estimated 6-year CVD risk (*P*-overall <0.001, *P*-nonlinear <0.001).

**Figure 2:**
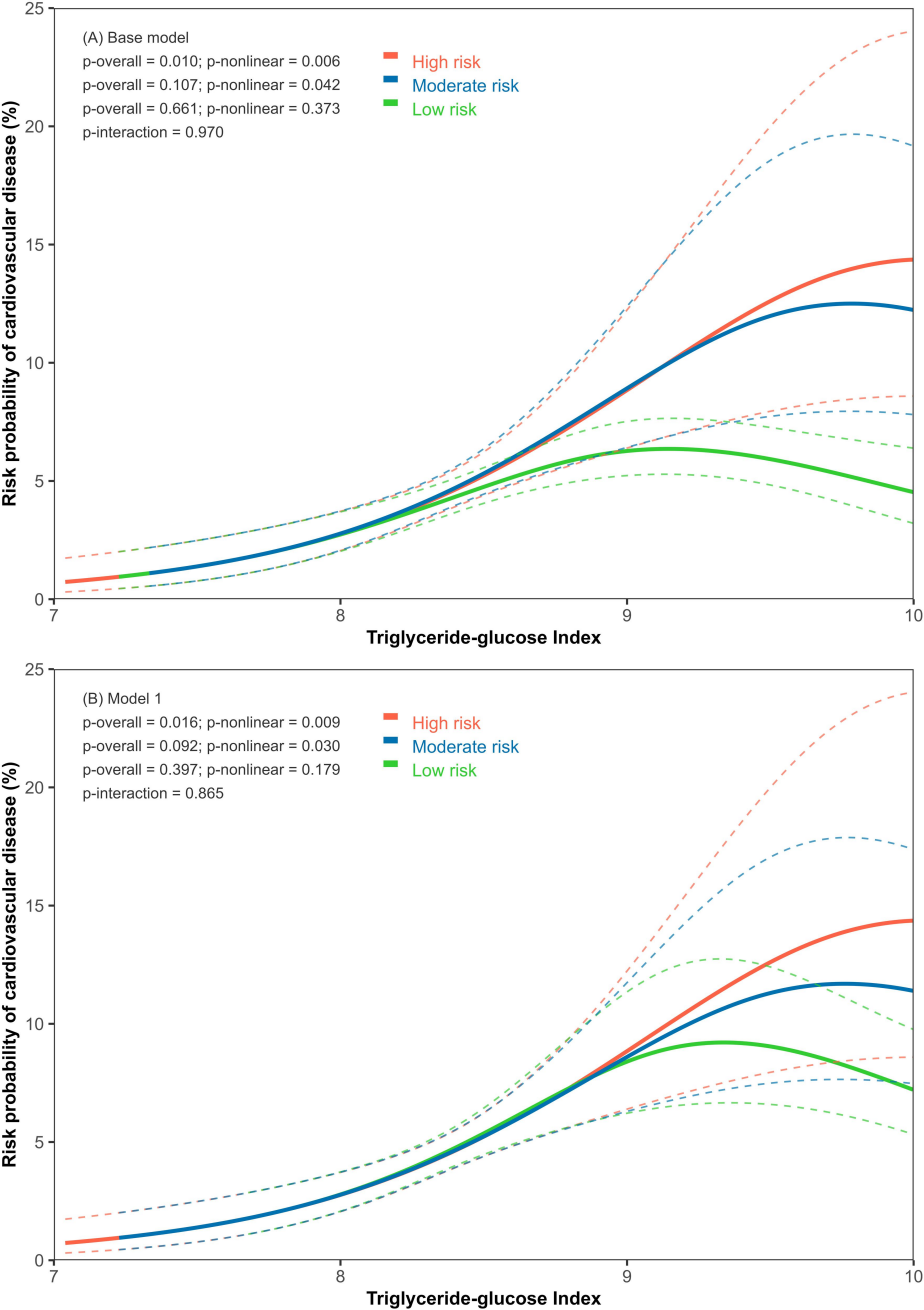
Flexible relationships between TyG index and risk probability of incident cardiovascular disease in different risk stratification. Participants were stratified into low-risk (<10%), intermediate-risk (10% to <20%), and high-risk (≥20%) categories based on predicted risk of base model and model 1. **(A)** Associations between TyG index and risk probability of CVD stratified by base model; **(B)** associations between TyG index and risk probability of CVD stratified by model1.

Figure 2 demonstrates that among high-risk individuals identified by both the base model and the model1, elevated TyG index exhibited the steepest association with CVD risk probability (both *P*-overall <0.05, *P*-nonlinearity <0.05), despite no significant interaction with risk stratification. Further subgroup analyses stratified by sex, age, hypertension history, diabetes history, alcohol consumption, smoking status, BMI, and Framingham risk categories showed no significant interactions with either continuous TyG index (Supplementary Figure S3) or TyG quartiles (Supplementary Table S5) (all *P*-interaction >0.05).

### 3.2 Meta-analysis

Database searches identified 5,318 records. After deduplication, 2,380 underwent title/abstract screening, and 45 full texts were assessed. Four cohort studies (13, 27–29) and the cohort study in this research met inclusion criteria for qualitative synthesis and meta-analysis. A detailed description of the search strategy is provided in Supplementary Table S6. CVD event incidence ranged from 3.3% (158/4,754) to 14.4% (1,084/7,521) across studies.

The total five studies included 27,329 participants (2,457 CVD events) from China (*n* = 2, this study contained) (29), the United States (*n* = 1) (13), Spain (*n* = 1) (28), and Iran (*n* = 1) (27), with sample sizes ranging from 4,665 to 7,521. Base models and outcomes are detailed in Table 3.

**Table 3:** Characteristics of included studies.

Source	Study name	Country	Study population	Racial and ethnic groups	Age, years	Risk model	Outcome	Individual outcomes included
Yu et al. (29)	The relationship between triglyceride glucose index and risk of cardiovascular disease among Kazakh and Uygur population in Xinjiang: a retrospective cohort analysis	China	No history of CVD; age > 18	NR (Chinese residents)	41.06 (30.11 to 53.00)	SCORE2-ASIA including age, sex, SBP, HDL, TC, smoking	CVD	Nonfatal and fatal CHD, stroke, and Peripheral vascular disease
Xu et al. (13)	High triglyceride-glucose index in young adulthood is associated with incident cardiovascular disease and mortality in later life: insight from the CARDIA study	US (Coronary Artery Risk Development in Young Adults study)	No history of CVD; age 18 to 30	Black and white in US	24.72 ± 3.62	PCEs model including age, race, sex, TC, HDL, SBP, diabetes, antihypertensive medication, and smoking	CVD	Nonfatal and fatal myocardial infarction, non-myocardial infarction acute coronary syndrome, stroke, coronary revascularization, transient ischemic attack, congestive heart failure, carotid or peripheral arterial disease requiring intervention
Barzegar et al. (27)	The impact of triglyceride-glucose index on incident cardiovascular events during 16 years of follow-up: Tehran Lipid and Glucose Study	Iran (Tehran Lipid and Glucose Study)	No history of CVD; age ≥ 30	NR (Iran resident)	46.6 ± 12.1	Framingham risk factors including age, gender, TC, HDL, SBP, antihypertensive drugs, smoking, and diabetes	CVD	CHD, fatal and nonfatal stroke
Sánchez-Íñigo et al. (28)	The TyG index may predict the development of cardiovascular events	Spain (The Vascular Metabolic CUN cohort)	No history of CVD; age 18 to 90	Caucasian population	55.51 ± 13.68 for women; 53.72 ± 12.84 for men	Framingham risk score model including age, sex, SBP, DBP, LDL, HDL, smoking and diabetes	CVD	CHD, cerebrovascular disease, and peripheral arterial disease
Liu et al. (2026)	Reassessing of the incremental predictive value of triglyceride-glucose index beyond traditional cardiovascular risk factors: a cohort study based on the china health and nutrition survey and meta-analysis	China (China Health and Nutrition Survey)	No history of CVD; age 45 to 80	NR (Chinese residents)	57.0 (51.0 to 64.0)	Framingham risk factors including age, sex, SBP, treatment of hypertension, TC, HDL, diabetes, and smoking	CVD	MI and stroke

As shown in Supplementary Figures S4 and S5, for study participation, most studies had a low risk of bias; 1 study (29) was rated as unclear due to insufficient description of the cluster sampling method. For attrition bias, all studies did not report reasons for loss to follow-up, characteristics of lost participants, or differences between completers and lost participants, thus rated as unclear. For prognostic factor measurement, all 5 studies had a low risk of bias. For outcome measurement: 2 studies (28, 29) had a low risk of bias, and 3 studies (this research included) (13, 27) were rated as unclear (details of outcomes in each study are listed in Table 3). For confounding factors: all studies used published CVD risk equations with established coefficients, indicating a low risk of bias in confounding control. For statistical analysis and reporting: 3 studies (this article involved) (13, 27) had a low risk of bias, and 2 studies (28, 29) were rated as unclear.

**Figure 3:**
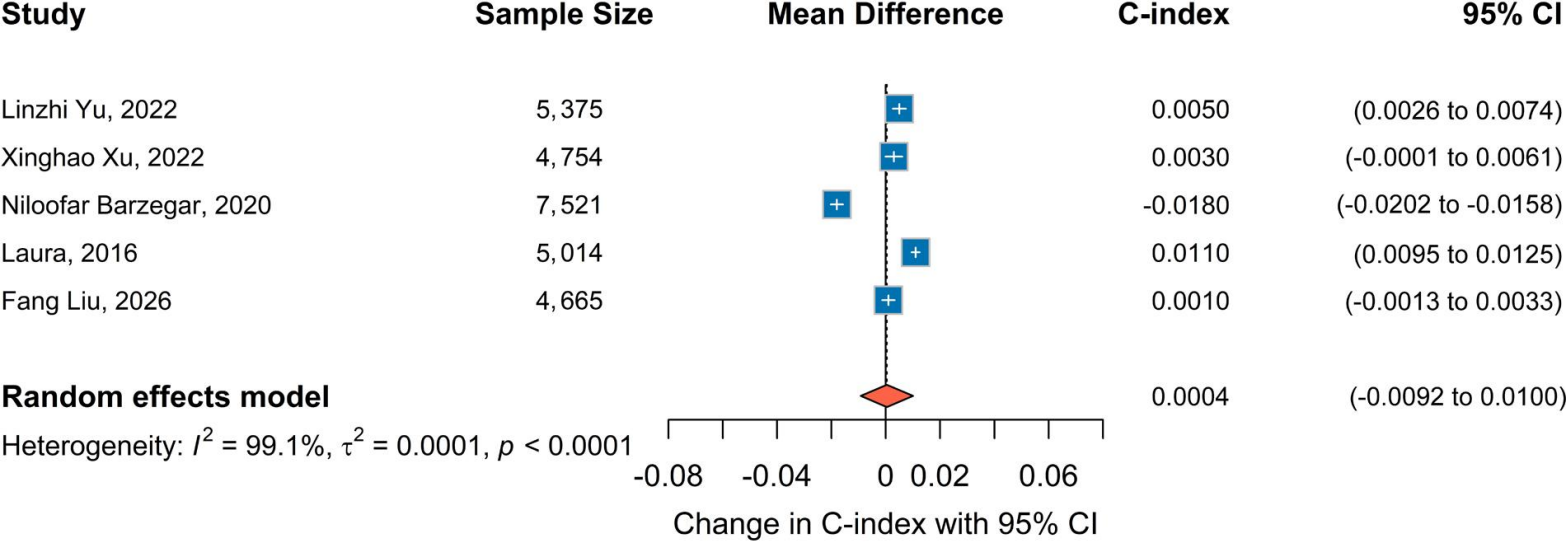
Meta-analysis of change in C-index with TyG index plus risk equation vs. risk equation alone.

C-index for CVD models without TyG index ranged from 0.708 (95% CI 0.68 to 0.73) to 0.789 (95% CI 0.773 to 0.805) (Table 4). The pooled C-index improvement was 0.0004 (95% CI −0.0092 to 0.0100) with substantial heterogeneity (*I*^2^ = 99.1%; Figure 3).

**Table 4:** Incremental value of TyG index to risk prediction models.

Source	No. of CV events/ total No. (%)	Length of follow up, years	Adjusted HR (95% CI)	Base model C-index (95% CI)	TyG model C-index (95% CI)	Reported change of C-index (95% CI)	Calculated change of C-index (95% CI)
Yu et al. (29)	500/5,375 (9.3%)	6.78 (4.78 to 6.79)	1.73 (1.27 to 2.37) for highest vs. lowest quartile	0.783 (0.763 to 0.804)	0.788 (0.768 to 0.808)	NR	0.0050 (0.0026 to 0.0074)
Xu et al. (13)	158/4,754 (3.3%)	A median of 25 years	3.67 (1.90 to 7.06) for highest vs. lowest quartile	0.773 (0.738 to 0.808)	0.776 (0.742 to 0.810)	NR	0.0030 (−0.0001 to 0.0061)
Barzegar et al. (27)	1,084/7,521 (14.4%)	16.1 (13.4 to 16.5)	1.61 (1.23 to 2.11) for highest vs. lowest quintile	0.789 (0.773 to 0.805)	0.771 (0.752 to 0.789)	NR	−0.0180 (−0.0202 to −0.0157)
Sánchez-Íñigo et al. (28)	505/5,014 (10.1%)	A median of 10 years	2.32 (1.65 to 3.26) for highest vs. lowest quintile	0·708 (0·68 to 0·73)	0·719 (0·70 to 0·74)	NR	0.0110 (0.0095 to 0.0125)
Liu et al. (2026)	210/4,665 (4.5%)	A median of 6 years	2.07 (1.23 to 3.49) for highest vs. lowest quartile	0.731 (0.698 to 0.764)	0.732 (0.699 to 0.765)	0.0010 (−0.0013 to 0.0033)	0.0010 (−0.0013 to 0.0033)

Leave-one-out sensitivity analysis (Supplementary Figure S6) indicated instability, primarily driven by Barzegar et al. (27), which reported an opposing effect size and had a large sample. Excluding this study yielded a statistically significant pooled C-index improvement of 0.0051 (95% CI 0.0007 to 0.0094). Heterogeneity may stem from regional variations (e.g., baseline CVD risk/genetic/religious factors). Additional subgroup analyses (Supplementary Figure S7A) showed that when restricted to 2 studies (with this cohort) (29) conducted in China, the gain in C-index was 0.0030 (95% CI: −0.0009 to 0.0069), which differed substantially from the overall pooled result. When restricted to 2 studies (with this study) (27) using FRS (Supplementary Figure S7B), the gain in C-index was −0.0085 (95% CI: −0.0027 to 0.0101), which was different from the overall pooled result. When restricted to 2 studies (28, 29) with outcomes including CHD, stroke, and peripheral vascular disease (Supplementary Figure S7C), the gain in C-index was 0.0081 (95% CI: 0.0022 to 0.0140).

As summarized in Supplementary Table S7, the overall certainty of evidence for the incremental C-index change in the main analysis was rated as very low. This rating was primarily driven by three downgrading factors: (i) serious risk of bias due to substantial attrition rates (ranging from 9.6% to 29%) across all included cohorts without systematic comparisons of lost participants; (ii) serious inconsistency reflected by extremely high statistical heterogeneity (*I*^2^ = 99.1%); and (iii) serious imprecision, as the pooled 95% confidence interval crossed the null value (−0.0092 to 0.0100). Although a dose-response relationship was observed across studies, we did not upgrade the evidence because the absolute effect size was clinically trivial. In the sensitivity analysis excluding the Barzegar et al. (27), the certainty of evidence remained very low. We maintained downgrades for persistent risk of bias and residual inconsistency due to differences in covariate adjustment and outcome definitions across the remaining four studies. The minimal effect size (ΔC = 0.0051) precluded upgrading despite the presence of a dose-response gradient.

## 4 Discussion

In the cohort study section, after adjusting for traditional cardiovascular risk factors, participants in the highest TyG index quartile had significantly higher risks of CVD and stroke compared to those in the lowest quartile (*P* < 0.01), and the association between the continuous TyG index and CVD incidence was non-linear. Secondly, adding continuous TyG index to the base model did not significantly improve the C-index, IDI, continuous NRI, and Categorial NRI were all non-significant, indicating negligible improvement in model discrimination and risk reclassification across low, intermediate, and high-risk categories. However, when TyG quartiles were incorporated, although the Delong test did not reach statistical significance, the IDI, continuous NRI, and event-group NRI all improved significantly, suggesting that the TyG quartile model may primarily enhance the identification of individuals who will eventually develop CVD, rather than improving the classification of those who will not.

A notable discrepancy emerged between the continuous and quartile-based TyG models regarding incremental predictive value. The RCS analysis (Poisson models) revealed a significant S-shaped non-linear relationship between TyG and the estimated 6-year CVD risk. Within this non-linear framework, the continuous TyG model, which enters TyG as a linear term in Cox regression, may inadequately capture the threshold effect at higher TyG levels, thereby attenuating its incremental performance. In contrast, the quartile-based model does not impose a linear trend and allows hazard ratios to vary across strata, effectively approximating the non-linear pattern. This flexibility likely explains its significant improvements in IDI and NRI, particularly in event reclassification. However, categorization-induced artifacts cannot be ruled out. The non-significant DeLong test (*P* = 0.2136) suggests that while the quartile model may improve reclassification in specific subgroups, it does not substantially enhance the global discriminative capacity of the FRS. Therefore, the clinical utility of TyG quartiles should be interpreted with caution, as the observed benefits may be partially driven by cut-point selection rather than a true biological threshold.

This meta-analysis included five cohort studies (this research included) (13, 27–29), showing variability in baseline population risk (CVD incidence: 3.3% to 14.4%). When comparing the CHNS cohort findings with the international meta-analysis results, the CHNS cohort showed a ΔC-index of 0.0010 for continuous TyG and 0.0080 for quartile-based TyG, both of which are broadly consistent with the overall pooled estimate of 0.0004. This alignment suggests that the limited incremental value observed in the Chinese population is generally reflective of the international evidence.

The advent of the Framingham Heart Study (30) and other large epidemiological cohorts has shifted the focus of CVD research from treating established illness to identifying high-risk individuals, enabling primary prevention through targeted interventions (31). However, the FRS and other risk instruments based on traditional risk factors remain prone to risk overestimation or underestimation (32). With the emergence of next-generation risk assessment tools, there is growing expectation that novel biomarkers can provide incremental, specific information beyond conventional risk factors, thereby enhancing the predictive capacity of existing risk assessment systems.

IR, a state characterized by reduced sensitivity of target organs to insulin, acts as a key driver of chronic inflammation, atherosclerosis, endothelial dysfunction, and oxidative stress (33), playing a crucial role in the development and progression of CVD (34). As an excellent marker for evaluating IR (35, 36), the TyG index has been validated by multiple high-quality clinical studies as a reliable predictor of CVD across diverse populations (37–39). Mendelian randomization analyses suggest a causal association between the TyG index and conditions such as HF (40) and hypertension (41). Previous meta-analyses (42) have shown that individuals in the highest TyG category have a higher incidence of CVD than those in the lowest (HR = 1.46, 95% CI: 1.23 to 1.74, *I*^2^ = 82%), although GRADE assessment indicates low certainty of evidence. In our meta-analysis, GRADE assessment likewise indicated very low certainty for the incremental value of TyG index, reinforcing the need for cautious interpretation. Beyond the four other studies included in this meta-analysis, several cohort studies using models with coefficients derived from cohort data have demonstrated that the TyG index confers only modest improvement in the predictive performance of baseline risk models for CVD incidence (43, 44).

This is the first meta-analysis to evaluate the incremental value of adding the TyG index to the conventional risk prediction models, yet the results exhibit extremely high heterogeneity. We propose the following potential sources: first, the set of traditional risk factors included in the baseline prediction models varied across studies. However, restricting the analysis to the two studies (this study contained) (27) that used FRS factors yielded estimates substantially different from the overall pooled effect, with no reduction in heterogeneity, suggesting this may not be the primary contributor. Second, outcome definitions differed across studies; when analyses were limited to the two studies (28, 29) with outcomes including CHD, stroke, and peripheral vascular disease, the result became statistically significant and heterogeneity decreased. Third, population characteristics varied. The sensitivity analysis excluding the Iranian study (27) led to a statistically significant pooled estimate among the remaining four studies. Of note, this study also had the highest baseline CVD incidence (14.4%) among all included cohorts, suggesting that the marked variation in baseline population risk (ranging from 3.3% to 14.4%) may be a major driver of heterogeneity. Other potential sources of heterogeneity include genetic background and age factors. Restricting the meta-analysis to the two Chinese studies (this study included) (29), reduced heterogeneity from 99.1% to 82%. Due to lack of age-specific data, we were unable to conduct age-stratified subgroup analyses. Further cohort studies are needed to explore the incremental value of the TyG index beyond traditional CVD risk factors across different age groups.

The innovation of this study lies in being the first to assess the incremental value of the TyG index over the FRS using data from the CHNS, with results incorporated into a meta-analysis. On the one hand, the cohort study revealed a significant non-linear association between the TyG index and future CVD events, providing new evidence for its utility as a predictor in middle-aged populations. On the other hand, both the cohort study and meta-analysis indicate that the incremental value of the TyG index for CVD risk prediction is limited, suggesting it may not enhance the predictive performance of established cardiovascular risk scores.

We hypothesize that the TyG index may refine risk stratification in specific subgroups. In the present cohort study, the TyG index showed limited reclassification capacity across risk strata. Novel methodologies and larger cohort studies are required to address current evidence gaps.

## 5 Limitations

However, the cohort study in this research has several limitations. First, the observational nature of the cohort study precludes definitive causal inference between the TyG index and CVD events. Second, despite adjustment for FRS, residual confounding may persist. Although this review performed comprehensive literature searches, bias assessments, and pooled analyses, potential limitations remain. First, participant attrition in included studies raises concerns about unclear risk of bias. Nevertheless, attrition likely affected both TyG index and traditional CVD risk factor measurements similarly, thus potentially having minimal impact on the estimation of incremental value. Second, only two risk prediction models—the FRS and the PCE—were used to evaluate the incremental value of CACS; the added benefit in models incorporating more variables (e.g., QRISK, PREDICT) remains unknown. Third, and importantly, the CHNS cohort is exclusively derived from the Chinese population, and our cohort analysis was restricted to middle-aged and older adults. Therefore, the findings of the cohort study may not be directly generalizable to younger populations, other ethnic groups, or different healthcare settings. Although the meta-analysis included studies from multiple countries (the United States, Spain, and Iran), the limited number of non-Chinese studies precludes robust cross-population comparisons. Consequently, our conclusions should be applied with caution to populations beyond the Chinese middle-aged and elderly demographic. Future studies across diverse populations are warranted to validate the incremental value of the TyG index in broader clinical practice.

## 6 Conclusion

In this combined cohort study and meta-analysis, while the TyG index showed a significant statistical association with incident CVD, its incremental predictive value beyond FRS factors was minimal at the cohort level and negligible at the meta-analytic level, with modest reclassification improvements failing to translate into meaningful gains in overall model discrimination. These findings, primarily derived from the CHNS cohort of middle-aged and elderly Chinese adults, should be generalized to other populations with caution and require external validation in younger or non-Asian cohorts. For the Chinese middle-aged and elderly population, we do not recommend routine incorporation of the TyG index into FRS-based risk assessment in clinical practice.

## Data Availability

The raw data supporting the conclusions of this article will be made available by the authors, without undue reservation.
