# Supplementary material for: Reassessing of the incremental predictive value of triglyceride-glucose index beyond traditional cardiovascular risk factors: a cohort study based on the China health and nutrition survey and meta-analysis
Source: Front Cardiovasc Med. 2026 Jul 22;13:1804411. doi: 10.3389/fcvm.2026.1804411 (PMC13449919; doi:10.3389/fcvm.2026.1804411)
Supplement: Supplementary file 1 [file Supplementaryfile1.docx]

**Table S1**

Parameter estimates and performance measures of the base model

| Parameter | HR (95% CI) |
| --- | --- |
| Age | 1.043(1.027-1.059) |
| Male sex | 1.526(1.101-2.116) |
| Systolic blood pressure | 1.019(1.012-1.026) |
| Treatment for hypertension | 2.020(1.468-2.781) |
| Total cholesterol | 1.153(1.007-1.320) |
| HDL cholesterol | 0.995(0.987-1.004) |
| Diabetes | 0.860(0.578-1.279) |
| Quit smoking | 1.411(0.784-2.540) |
| Never smoking | 1.148(0.800-1.647) |

**Table S2**

Associations of TyG index and TyG quartiles with CVD, MI and stroke incidence

| TyG index |  | | Unadjusted | | | Base model | |
| --- | --- | --- | --- | --- | --- | --- | --- |
|  | Incident CVD | HR (95%CI) | | P | HR (95%CI) | | P |
| Continues | 210/4,665 | 1.259 (1.050-1.510) | | 0.019 | 1.063 (0.833-1.356) | | 0.624 |
| Quartiles 1 | 25/1,168 | Ref | |  | Ref | |  |
| Quartiles 2 | 56/1,165 | 2.276 (1.420-3.646) | | 0.001 | 2.145 (1.329-3.461) | | 0.002 |
| Quartiles 3 | 65/1,166 | 2.648 (1.670-4.200) | | ＜0.001 | 2.169 (1.337-3.519) | | 0.002 |
| Quartiles 4 | 64/1,166 | 2.612 (1.645-4.148) | | ＜0.001 | 2.073 (1.233-3.488) | | 0.006 |

| TyG index |  | | Unadjusted | | | Base model | |
| --- | --- | --- | --- | --- | --- | --- | --- |
|  | Incident MI | HR (95%CI) | | P | HR (95%CI) | | P |
| Continues | 92/4,665 | 1.250 (0.949-1.646) | | 0.113 | 0.855 (0.580-1.260) | | 0.430 |
| Quartiles 1 | 13/1,168 | Ref | |  | Ref | |  |
| Quartiles 2 | 27/1,165 | 2.096 (1.082-4.063) | | 0.028 | 1.729 (0.877-3.407) | | 0.114 |
| Quartiles 3 | 23/1,166 | 1.786 (0.905-3.526) | | 0.095 | 1.176 (0.566-2.446) | | 0.664 |
| Quartiles 4 | 29/1,166 | 2.261 (1.176-4.350) | | 0.015 | 1.235 (0.559-2.729) | | 0.601 |

| TyG index |  | | Unadjusted | | | Base model | |
| --- | --- | --- | --- | --- | --- | --- | --- |
|  | Incident stroke | HR (95%CI) | | P | HR (95%CI) | | P |
| Continues | 126/4,665 | 1.327 (1.053-1.673) | | 0.017 | 1.223 (0.918-1.629) | | 0.168 |
| Quartiles 1 | 13/1,168 | Ref | |  | Ref | |  |
| Quartiles 2 | 29/1,165 | 2.254 (1.172-4.337) | | 0.015 | 2.240 (1.158-4.333) | | 0.017 |
| Quartiles 3 | 44/1,166 | 3.436 (1.851-6.379) | | ＜0.001 | 3.018 (1.595-5.712) | | 0.001 |
| Quartiles 4 | 40/1,166 | 3.121 (1.669-5.835) | | ＜0.001 | 2.807 (1.428-5.521) | | 0.002 |

TyG: triglyceride-glucose; CVD: cardiovascular disease; MI: myocardial infraction; FRS: Framingham risk score; HR: hazard ratio;

Base model: adjusted for age, sex, systolic blood pressure, antihypertensive treatment status, total cholesterol, high-density lipoprotein cholesterol, current smoking status, and diabetes

**Table S3**

Cardiovascular Disease Risk Reclassification After Extending the Base Model With TyG index

| FRS + TyG | FRS Alone | | | Row Total※ | Categorial NRI (95% CI) | P |
| --- | --- | --- | --- | --- | --- | --- |
|  | <10% | 10%-20% | >20% |  |  |  |
| Event (n = 210) |  | | | | -0.010(-0.029 to 0.009) | 0.320 |
| < 10% | 144 | 1 | 0 | 126 (60.0%) |  |  |
| 10% to 20% | 9 | 47 | 0 | 56 (26.7%) |  |  |
| > 20% | 0 | 1 | 15 | 16 (7.6%) |  |  |
| Column total | 153 (72.9%) | 49 (23.3%) | 15 (7.14%) |  |  |  |
| Nonevent (n = 4455) |  | | | | 0.001 (-0.001 to 0.004) | 0.385 |
| < 10% | 4227 | 15 | 0 | 4,242 (95.2%) |  |  |
| 10% to 20% | 15 | 339 | 0 | 354 (7.9%) |  |  |
| > 20% | 0 | 7 | 62 | 69 (1.5%) |  |  |
| Column total | 4242 (95.2%) | 361 (8.1%) | 62 (1.4%) |  |  |  |
| Total NRI for TyG |  | | | | -0.008 (-0.028 to 0.010) | 0.384 |

TyG: Triglyceride-glucose; FRS: Framingham risk score; NRI: Net reclassification improvement;

※Numbers (percentages) of persons with or without an event who are reclassified to a higher or lower risk category after the base model is extended with CAC score.

**Table S4**

Cardiovascular Disease Risk Reclassification After Extending the Base Model With TyG quartiles

| FRS + TyG | FRS Alone | | | Row Total※ | Categorial NRI (95% CI) | P |
| --- | --- | --- | --- | --- | --- | --- |
|  | < 10% | 10% to 20% | >20% |  |  |  |
| Event (n = 210) |  | | | | 0.0476 (0.0048 to 0.0913) | 0.0316 |
| < 10% | 136 | 9 | 0 | 145 (69.0%) |  |  |
| 10% to 20% | 3 | 39 | 7 | 49 (23.3%) |  |  |
| > 20% | 0 | 3 | 13 | 16 (7.6%) |  |  |
| Column total | 139 (66.2%) | 51 (24.3%) | 20 (9.5%) |  |  |  |
| Nonevent (n = 4455) |  | | | | -0.0007 (-0.0058 to 0.0045) | 0.7997 |
| < 10% | 4,040 | 57 | 0 | 4,097 (92.0%) |  |  |
| 10% to 20% | 59 | 232 | 14 | 305 (6.8%) |  |  |
| > 20% | 0 | 9 | 44 | 53 (1.2%) |  |  |
| Column total | 4,099 (92.0%) | 298 (6.7%) | 58 (1.3%) |  |  |  |
| Total NRI for TyG |  | | | | 0.0469 (0.0033 to 0.0906) | 0.0349 |

TyG: Triglyceride-glucose; FRS: Framingham risk score; NRI: Net reclassification improvement;

※Numbers (percentages) of persons with or without an event who are reclassified to a higher or lower risk category after the base model is extended with CAC score.

**Table S5**

Associations of the TyG quartiles with cardiovascular stratified by different factors

| CVD | TyG index, HR (95%CI) | | | | P for trend | P for interaction |
| --- | --- | --- | --- | --- | --- | --- |
|  | Quartile 1 (7.02 to 8.22) | Quartile 2 (8.22 to 8.64) | Quartile 3 (8.64 to 9.13) | Quartile 4 (9.13 to 12.26) |  |  |
| Sex |  |  |  |  |  | 0.870 |
| Male | Ref | 2.064 (1.114 to 3.825) | 2.318 (1.241 to 4.329) | 1.958 (0.994 to 3.854) | 0.058 |  |
| Female | Ref | 2.272 (1.057 to 4.885) | 2.044 (0.942 to 4.433) | 2.244 (0.980 to 5.137) | 0.193 |  |
| Age |  |  |  |  |  | 0.131 |
| Age < 55 | Ref | 1.911 (0.480 to 7.598) | 2.113 (0.545 to 8.199) | 4.553 (1.313 to 15.790) | 0.030 |  |
| 55 ≤ Age < 65 | Ref | 1.621 (0.737 to 3.567) | 1.758 (0.807 to 3.829) | 1.328 (0.558 to 3.157) | 0.446 |  |
| 65 ≤ Age | Ref | 1.593 (0.777 to 3.262) | 2.267 (1.132 to 4.540) | 2.300 (1.083 to 4.884) | 0.102 |  |
| Hypertension |  |  |  |  |  | 0.870 |
| Yes | Ref | 2.556 (1.283 to 5.088) | 2.644 (1.345 to 5.195) | 2.384 (1.179 to 4.822) | 0.036 |  |
| No | Ref | 1.517 (0.765 to 3.007) | 1.254 (0.598 to 2.631) | 1.134 (0.491 to 2.621) | 0.630 |  |
| Diabetes |  |  |  |  |  | 0.968 |
| Yes | Ref | 1.362 (0.153 to 12.115) | 1.701 (0.202 to 14.299) | 1.681 (0.206 to 13.697) | 0.943 |  |
| No | Ref | 2.103 (1.284 to 3.446) | 2.041 (1.232 to 3.381) | 1.921 (1.102 to 3.347) | 0.022 |  |
| Alcohol Consumption |  |  |  |  |  | 0.234 |
| Yes | Ref | 1.417 (0.537 to 3.737) | 2.096 (0.899 to 4.884) | 2.546 (1.167 to 5.533) | 0.070 |  |
| No | Ref | 1.972 (1.068 to 3.641) | 2.343 (1.281 to 4.286) | 2.535 (1.338 to 4.801) | 0.030 |  |
| Tobacco use |  |  |  |  |  | 0.278 |
| Current | Ref | 2.114 (0.678 to 6.590) | 4.214 (1.552 to 11.441) | 3.259 (1.192 to 8.912) | 0.018 |  |
| Never | Ref | 1.831 (1.040 to 3.221) | 1.550 (0.866 to 2.773) | 1.942 (1.059 to 3.560) | 0.135 |  |
| Quit | Ref | 1.667 (0.166 to 16.777) | 2.300 (0.218 to 24.300) | 1.738 (0.141 to 21.391) | 0.910 |  |
| BMI |  |  |  |  |  | 0.082 |
| BMI < 24 | Ref | 2.371 (1.218 to 4.615) | 2.581 (1.289 to 5.167) | 2.502 (1.158 to 5.407) | 0.043 |  |
| 24 ≤ BMI < 28 | Ref | 1.267 (0.583 to 2.754) | 1.048 (0.483 to 2.274) | 1.082 (0.470 to 2.490) | 0.907 |  |
| 28 ≤ BMI | Ref | 2.198 (0.432 to 11.173) | 2.246 (0.466 to 10.823) | 1.561 (0.312 to 7.813) | 0.681 |  |
| Framingham Risk category |  |  |  |  |  | 0.983 |
| < 10% | Ref | 2.048 (1.193 to 3.515) | 2.033 (1.164 to 3.550) | 1.845 (1.014 to 3.358) | 0.052 |  |
| 10% to 20% | Ref | 3.722 (1.011 to 13.705) | 3.966 (1.069 to 14.722) | 4.685 (1.139 to 19.281) | 0.183 |  |
| > 20% | Ref | 2.350 (0.266 to 20.740) | 1.303 (0.238 to 7.122) | 1.722 (0.282 to 10.533) | 0.864 |  |

TyG: Triglyceride-glucose; CVD: cardiovascular disease; HR: hazard ratio; BMI: body mass index;

Adjusted for age, sex, systolic blood pressure, antihypertensive treatment status, total cholesterol, high-density lipoprotein cholesterol, current smoking status, and diabetes

**Table S****6**

Detailed description of the search strategy

| PubMed | |
| --- | --- |
| #1 | ‘cardiovascular disease’ [MeSH Terms] |
| #2 | ("search"[All Fields] OR "searched"[All Fields] OR "searches"[All Fields] OR "searching"[All Fields] OR "searchs"[All Fields]) AND "heart"[Title/Abstract] |
| #3 | "coronar*"[Title/Abstract] OR "cardiac*"[Title/Abstract] OR "cardio"[Title/Abstract] OR "cardiog*"[Title/Abstract] OR "cardiol*"[Title/Abstract] OR "cardiom*"[Title/Abstract] OR "cardiop*"[Title/Abstract] OR "cardiov*"[Title/Abstract] OR "myocardi*"[Title/Abstract] OR "vascular*"[Title/Abstract] OR "CVD"[Title/Abstract] OR "cerebrovascular"[Title/Abstract] OR "stroke"[Title/Abstract] OR "cerebral"[Title/Abstract] OR "atheroscler*"[Title/Abstract] |
| #4 | #1 OR #2 OR #3 |
| #5 | "TyG index"[Title/Abstract] OR "triglyceride-glucose index"[Title/Abstract] OR "triglyceride and glucose index"[Title/Abstract] OR "triglyceride-glucose index"[Title/Abstract] OR "triacylglycerol glucose index"[Title/Abstract] OR "TyG"[Title/Abstract] |
| #6 | #4 AND #5 |
| Embase | |
| #1 | ‘cardiovascular disease’:ab,ti |
| #2 | ('search' OR 'searched' OR 'searches' OR 'searching' OR 'searchs') AND 'heart':ab,ti |
| #3 | ‘coronar*’:ab,ti OR ‘cardiac*’:ab,ti OR ‘cardio’:ab,ti OR ‘cardiog*’:ab,ti OR ‘cardiol*’:ab,ti OR ‘cardiom*’:ab,ti OR ‘cardiop*’:ab,ti OR ‘cardiov*’:ab,ti OR ‘myocardi*’:ab,ti OR ‘vascular*’:ab,ti OR ‘CVD’:ab,ti OR ‘cerebrovascular’:ab,ti OR ‘stroke’:ab,ti OR ‘cerebral’:ab,ti OR ‘atheroscler*’:ab,ti |
| #4 | 'tyg index':ab,ti OR 'triglyceride and glucose index':ab,ti OR 'triglyceride-glucose index':ab,ti OR 'triacylglycerol glucose index':ab,ti OR 'tyg':ab,ti |
| #5 | #1 OR #2 OR #3 |
| #6 | #4 OR #5 |
| Cochrane | |
| #1 | cardiovascular disease OR coronar* OR cardiac* OR cardio OR cardiog* OR cardiol* OR cardiom* OR cardiop* OR cardiov* OR myocardi* OR vascular* OR CVD OR cerebrovascular OR stroke OR cerebral OR atheroscler* |
| #2 | (search OR searched OR searches OR searching OR searchs) AND heart |
| #3 | "TyG index" OR "triglyceride-glucose index" OR "triglyceride and glucose index" OR "triglyceride-glucose index" OR "triacylglycerol glucose index" OR TyG |
| #4 | #1 OR #2 |
| #5 | #5 OR #6 |

**Table S7**

GRADE evidence profile for the incremental predictive value of the TyG index for cardiovascular disease

| **№ of studies** | **Study design** | **Risk of bias** | **Inconsistency** | **Indirectness** | **Imprecision** | **Other considerations** | **Effect (95% CI)** | **Certainty** |
| --- | --- | --- | --- | --- | --- | --- | --- | --- |
| Change in C-index (n = 5) | observational cohort studies | serious^a^ | serious^b^ | not serious | serious^c^ | none^d^ | 0.0004 (-0.0092 to 0.0100) | very low |
| Sensitivity analysis excluding Niloofar Barzegar et al, 2020 (n = 4) | observational cohort studies | serious^e^ | serious^f^ | not serious | not serious | none^g^ | 0.0051 (0.0007 to 0.0094) | very low |

Explanations:

a. All included cohort studies had substantial attrition rates (ranging from 9.6% to 29%), and none provided a systematic comparison of baseline characteristics between completers and those lost to follow‑up. Although the TyG index was objectively measured in central laboratories and multivariable adjustments were performed, the potential for selection bias due to incomplete follow‑up cannot be excluded; therefore, we downgraded by one level for serious risk of bias.

b. The meta‑analysis showed extremely high statistical heterogeneity, and the direction of effect estimates was inconsistent: four studies reported positive increments in the C‑statistic, whereas the study by Niloofar Barzegar et al. (2020) showed a negative change. This inconsistency, largely driven by a single outlier, was judged to be serious, warranting a one‑level downgrade.

c. The pooled 95% confidence interval for the change in C‑statistic crossed the null value (from -0.0092 to 0.0100), indicating that the estimate is compatible with both no improvement and a trivial improvement; we therefore downgraded one level for imprecision.

d. Although a clear dose‑response relationship was observed across our studies, the absolute increment in the C‑statistic was extremely small (ΔC = 0.0004). Given this minimal effect magnitude and the substantial heterogeneity, we did not upgrade the evidence.

e. The four remaining cohort studies still had high attrition rates (9.6%–29%) and failed to adequately report the characteristics of participants lost to follow‑up; thus, we maintained a one‑level downgrade for serious risk of bias.

f. Even after excluding the outlier study, moderate‑to‑substantial heterogeneity persisted across the remaining studies, mainly due to differences in covariate adjustment sets and outcome definitions. We consequently downgraded one level for inconsistency.

g. A statistically significant dose‑response gradient was present (P for trend <0.001), but the incremental improvement in the C‑statistic remained very small (ΔC = 0.0051) and of limited clinical relevance. Accordingly, we did not upgrade the evidence qua

9,537 Participants aged in China Health and Nutrition Survey at baseline wave 2009

5,159 participants at baseline were followed up from 2009 to 2015

3,996 participants < 45 years old

155 participants > 80 years old

206 participants with prevalent cardiovascular disease at baseline

21 participants without TyG index

494 participants without proper followed up information

4,665 participants were finally included

in the analysis

**Figure S1.** enrollment flowchart for the cohort study.

Records identified from databases (n = 5,318, including PubMed = 1,437, Embase = 2,093, Web of Science = 1,788)

Records removed before screening: duplicate records removed (n = 2,938)

Reports assessed for eligibility (n = 45)

Reports excluded (n = 41)

Absence or inappropriate basic model (n = 24)

Lack of essential statistical methods (n = 8)

Other outcomes (n = 5)

Population (n = 2)

Study type (n = 1)

Records excluded based on titles or abstracts (n = 2,228)

Study included (n = 4)

Records screened (n = 2,380)

**Figure S2.** enrollment flowchart for the meta analysis.

**Figure S3.** Associations of the TyG index with cardiovascular stratified by different factors

TyG: Triglyceride-glucose; CVD: cardiovascular disease; HR: hazard ratio; BMI: body mass index;

Adjusted for age, sex, systolic blood pressure, antihypertensive treatment status, total cholesterol, high-density lipoprotein cholesterol, current smoking status, and diabetes

**Figure S4.** Risk of Bias of Included Studies

**Figure S5** Risk of Bias for Included Studies

Legend: D1=Study participation; D2=Study attrition; D3=Prognostic factor measurement; D4=Outcome measurement; D5=Study confounding; D6=Statistical analysis and reporting. Yellow(-)=Unclear risk of bias; Green(+)=Low risk of bias

**Figure S6.** The pooled effect size of all the remaining studies after excluding every study

**Figure S7.** (A) Restricted to studies that in the Chinese population; (B) Restricted to studies that use Framingham risk factors; (C) Restricted to studies that outcome including CHD, stroke and peripheral arterial disease
